# A biocompatible cellulose gum based CMC/PVA/SBA-15 film as a colloidal antibacterial agent against MRSA†

**DOI:** 10.1039/d4ra07129h

**Published:** 2024-11-13

**Authors:** Shiva Pakzad, Reza Taghavi, Amir Hasanzadeh, Sadegh Rostamnia

**Affiliations:** a Cellular and Molecular Research Center, Cellular and Molecular Medicine Research Institute, Urmia University of Medical Sciences Urmia 57157-89400 Iran hasanzadeh.a@umsu.ac.ir; b Organic and Nano Group, Department of Chemistry, Iran University of Science and Technology Tehran 16846-13114 Iran rostamnia@iust.ac.ir srostamnia@gmail.com

## Abstract

The development of biocompatible antibacterial films plays a crucial role in the fight against antibiotic-resistant bacteria strains. Here, we developed an SBA-15–NH_2_ decorated biocompatible CMC/PVA film containing Ag NPs as an antibacterial material against Gram-positive and Gram-negative bacteria strains. The structure of the manufactured film was studied by XRD, SEM, mapping, and TGA analysis showing its formation and firm structure. The prepared film has a flexible structure which makes it suitable for a variety of bio-related applications. The CMC/PVA/SBA-15–NH_2_@AgNPs film was used as a bactericidal agent against pathogens (especially MRSA; methicillin-resistant *Staphylococcus aureus*) isolated from surgical site infections, showing promising results.

## Abstract 2

The development of biocompatible antibacterial films plays a crucial role in the fight against antibiotic-resistant bacteria strains.

## Graphical abstract

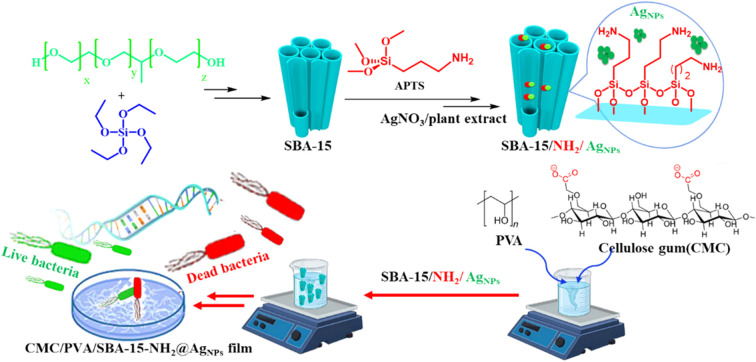

## 1. Introduction

Surgical site infections (SSIs) are common nosocomial infections and are associated with high morbidity and mortality.^1^ In addition, the emergence of high antimicrobial resistance among bacterial pathogens has made the management of postoperative wound infection difficult.^1,2^ Therefore, due to the high resistance of these bacteria, it is necessary to use alternative drugs that can eliminate these resistant bacteria.^3^ Nowadays, one of the things that is mostly discussed as an antibacterial substance is nanoparticles stabilized into a biocompatible support.^4,5^

Employing carboxymethyl cellulose (CMC) and polyvinyl alcohol (PVA) as precursor polymers for the production of biodegradable films is a well-established methodology both in the laboratory and industry. The CMC–PVA film is known for its remarkable properties, such as high tensile strength, flexibility, and water solubility.^6^ Such properties make this film an ideal candidate for packaging, agriculture, and pharmaceutical applications.^7,8^ The CMC component imparts water-binding and viscosity-enhancing capabilities, while PVA contributes to the film's mechanical strength and film-forming properties. This combination results in a versatile material that is not only environmentally friendly but also customizable in terms of its physical and chemical characteristics.^9–11^

SBA-15 is a mesoporous silica-based material with a unique hexagonal pore structure that is well known for its high surface area and homogeneous pore size distribution. SBA-15's porous porosity and structured structure provide a flexible substrate with a large surface area, making it a good choice for metal nanoparticle stabilization and heterogenization.^12,13^ Furthermore, SBA-15's capacity to be functionalized with various additions makes it vulnerable to a variety of situations and uses.^14,15^ Furthermore, it can easily create composites with other materials, which aids its ever-expanding uses in numerous fields of research.^16–18^

The unique physicochemical characteristics of silver nanoparticles (Ag NPs), such as small size, high surface area, and intrinsic properties cause their antibacterial properties.^19^ The small size of the Ag NPs results in an increased surface area, enhancing contact with bacterial cells and release of silver ions that disrupt essential cellular processes.^20,21^ This disruption compromises cell integrity, inhibits growth, and triggers oxidative stress, leading to bacterial cell death. The versatile nature of Ag NPs enables their incorporation into various materials, such as coatings, textiles, and medical devices, imparting antimicrobial activity.^22,23^

**Scheme 1:**
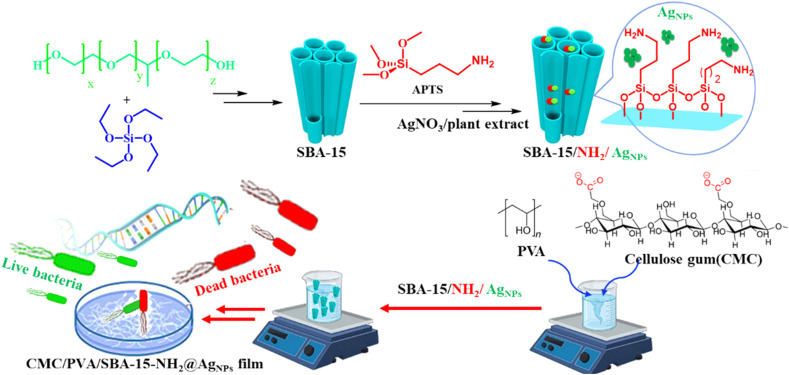
Schematic illustration of the CMC/PVA/SBA-15–NH_2_@AgNPs film preparations and bactericidal agent against pathogens of MRSA.

In this study, we prepared an antibacterial SBA-15–NH_2_-adorned biocompatible CMC/PVA film containing Ag NPs against Gram-positive and Gram-negative bacteria strains. The structure of the created film was analyzed using FT-IR, XRD, SEM, EDS, and TGA techniques to reveal its structure. Due to its flexible structure, the fashioned film is ideal for a wide range of bio-related applications. The CMC/PVA/SBA-15–NH_2_@AgNPs film was tested as a bactericidal agent against bacteria isolated from surgical site infections, and the findings were encouraging (Scheme 1).

## 2. Experimental

### 2.1. Materials

All the materials and reagents were prepared from commercial sources and were used without any further purification.

### 2.2. Preparation of SBA-15

4 g of P123 surfactant was dissolved in 2 N hydrochloric acid and agitated for 24 hours to create micelles in mesoporous materials. After confirming that all surfactants had been formed, the temperature of the mixture was elevated to 40 °C to produce a tubular aggregation of round or spherical micelles. Next, 9 g of tetraethyl orthosilicate (TEOS) was added to the mixture dropwise and agitated for 24 hours at 40 °C on a stirrer. The gel was placed in a steel autoclave and held at 100 °C for 24 hours. The finished product was rinsed with water and ethanol many times. Finally, the material was dried in an oven before being calcined in a temperature-regulating furnace at 550 °C with a temperature gradient of 1 °C min^−1^.

### 2.3. Amin functionalization of SBA-15

For this purpose, 1 g of calcined SBA-15 was completely dispersed in 100 mL of xylene, and 1 mmol of 3-(triethoxysilane)propylamine (APTS) was added dropwise to the mixture at room temperature while stirring. After homogenization of the mixture, it was refluxed for 12 hours at 90 °C for hydrolysis of the corresponding APTS inside the mesoporous cavities of SBA-15. The resulting solid was filtered, washed with ethanol, and dried at 60 °C.

### 2.4. Collection of the *Euphorbia* plant extract

The aerial portions of the *Euphorbia* plant were harvested in the full flowering stage in the Maragheh district of Iran and dried in the shade. The appropriate *Euphorbia* plants' green branches were then pulverized into powder at room temperature. The maceration method is used for extraction, in which 100 g of plant powder is weighed and placed within a beaker, and then 99.7% ethanol is poured to cover the plant. It was filtered using filter paper and a funnel after 24 hours, and the process was done three times. Finally, the ethanol was evaporated and the extract was separated.

### 2.5. Stabilization of Ag NPs over the surface of SBA-15–NH_2_

First, 3 mL of distilled water was used to dissolve 0.004 g of silver nitrate, which was then added to 10 mL of deionized water containing 0.1 g of SBA-15. The resultant liquid was swirled for one hour on the stirrer before adding the *Euphorbia* plant extract and stirring for another hour. It was washed, centrifuged, and dried at 60 °C.

### 2.6. Preparation of CMC/PVA/SBA-15–NH_2_@AgNPs film

First, a homogeneous solution of carboxymethyl cellulose (CMC) was prepared by dissolving CMC in deionized water at 70 °C under magnetic stirring. Similarly, polyvinyl alcohol (PVA) solution was prepared by adding PVA to deionized water at 70 °C and stirring for three hours. Finally, the SBA-15–NH_2_@AgNPs was added to the above mixture under stirring to prepare the desired nanocomposite. Finally, the desired nanocomposite was prepared using ultrasonication for 30 minutes. The mixture was poured into a clean watch glass and dried at room temperature. The final product is CMC/PVA/SBA-15@AgNPs nanocomposites in the form of a film. Separation from the container is investigated to check the amount of absorption of metal ions in the aqueous environment.

### 2.7. Study design

In this study, tests were performed on 20 bacteria isolated from patients with surgical wound infection. The isolates were collected from Urmia Medical Science Hospital laboratory and confirmed by additional tests. After making the nanofilm and isolating the bacteria, the cytotoxicity and antibacterial properties of the nanofilm were evaluated. Each experiment was repeated three times, and statistical analysis was performed with SPSS Version 21. The results were presented as mean ± standard deviation, and *P* ≤ 0.05 was considered statistically significant.

### 2.8. Properties of antibacterial and anti-biofilm nanofibers

The assessment of the antibacterial properties of nanofibers involves applying the disc diffusion method to standard and isolated bacterial strains. Initially, bacterial isolates were cultured on nutrient agar. After 24 hours, several bacterial colonies were suspended in physiological serum to achieve a concentration of 1.5 × 10^8^ CFU mL^−1^. The bacterial suspension was then spread on Muller–Hinton agar using the lawn culture method. Subsequently, 5 mm diameter discs cut from nanofibers containing various silver nanoparticle concentrations (3%, 7%, and 10%) were placed on the agar surface for 15 minutes. The cultures were incubated at 37 °C for 24 hours, and after incubation, the inhibition zone around the discs was examined and measured. Ciprofloxacin antibiotic discs were used as a control for comparison, and all experiments were replicated three times.

The anti-biofilm impact of nanofibers was studied using different silver nanoparticle concentrations (3%, 7%, and 10%) on standard Gram-positive (*Staphylococcus aureus*; ATCC-43300) and Gram-negative (*Pseudomonas aeruginosa*; ATCC-27853) strains through the microtiter plate technique and safranin staining. In summary, 5 mm diameter nanofiber discs were positioned in wells, and 100 μL of TSB medium (comprising 5% glucose and a bacterial suspension with a final concentration of 4 × 10^5^ CFU mL^−1^) was introduced into the wells. After three PBS washes, the well contents were fixed with 100 μL of a 0.1% crystal violet solution, stabilizing the cells. Subsequently, 100 μL of ethanol was introduced into the wells, and after a 5 minute lapse, the optical absorption of the stained wells was measured at a wavelength of 492 nm using a microplate reader.

### 2.9. Cell culture and cytotoxicity assessment

Cell culture and investigation of cytotoxicity properties involved cultivating Primary Normal Human Dermal Fibroblasts (NHDF) in RPMI medium supplemented with 20% fetal bovine serum and a 1% antibiotic solution (penicillin–streptomycin) under controlled conditions with 95% humidity, 5% CO_2_, and a temperature maintained at 37 degrees Celsius. PVA/CMC nanofibers with or without silver nanoparticle content were cut into 5 mm diameter discs and sterilized under ultraviolet light for 1 hour on both sides. The discs were washed with sterile PBS and placed in the wells of a 96-well plate. Following trypsinization and centrifugation, the cultured cells were suspended in a fresh culture medium. Then, 150 μL of the cell suspension, with a concentration of 3 × 10^4^, was introduced into each well-containing nanofiber. The plate was subsequently incubated for 24 hours. The MTT assay was used to evaluate cell viability. After discarding the old culture medium, cells were washed once with PBS. Afterward, 100 μL of a fresh medium (containing MTT solution 200 mg mL^−1^) was introduced into each well, and the plate underwent incubation at 37 °C for 4 hours. Afterward, dimethyl sulfoxide was added to the wells; after a 30 minute duration at room temperature, the optical density of viable cells was measured at 570 nm using an ELISA microplate reader.

## 3. Results and discussion

**Scheme 2:**
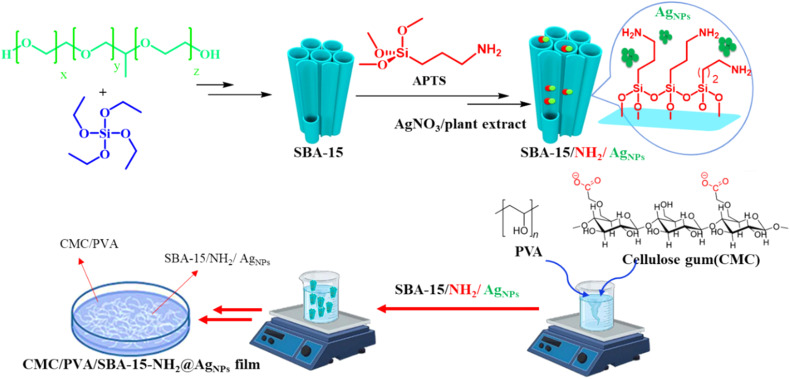
Schematic illustration of the preparation of the CMC/PVA/SBA-15–NH_2_@AgNPs film.

In order to prepare an appropriate platform for the stabilization of silver NPs, the bio-compatible film was prepared through a step-by-step strategy. First, SBA-15 was prepared through a solvothermal method, and a post-synthesis modification strategy was hired for its functionalization with amine groups. Next, a wet impregnation method was employed for the stabilization of Ag NPs into the pores of the SBA-15 to prepare SBA-15s with 3, 7, and 10 percent of Ag content. The presence of amine functionalities facilitates the impregnation process and results in monodispersity of Ag NPs in the SBA-15 structure. Finally, the Ag NPs impregnated SBA-15–NH_2_ were added to the synthesis environment of the CMC–PVA to prepare the final film. The as-prepared biocompatible CMC/PVA/SBA-15@AgNPs film was used as an antibacterial agent against Gram-positive and Gram-negative bacteria (Scheme 2).

**Fig. 1:**
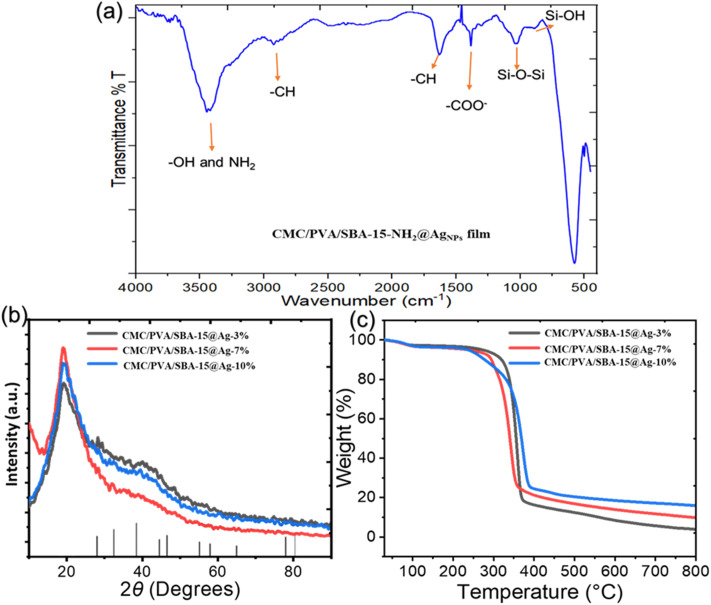
(a) The FT-IR analysis of the CMC/PVA/SBA-15–NH_2_@AgNPs. (b) XRD patterns of the CMC/PVA/SBA-15–NH_2_@AgNPs films, (c) the TGA plots of the CMC/PVA/SBA-15–NH_2_@AgNPs films.

The FT-IR spectra of the CMC/PVA/SBA-15–NH_2_@AgNPs film is represented in Fig. 1a. The peaks at 802 and 968 cm^−1^ represent the presence of the Si–OH and Si–O–Si, respectively. The presence of the sharp peaks at 1086 further proves the presence of these bondings. Moreover, a peak around 1377 and 2930 cm^−1^ is appeared which regards the presence of the C–H groups of the APTES and the CMC/PVA film. The surface –OH groups of the CMC/PVA film, the SBA-15, and the attached –NH_2_ groups to the SBA-15 is appeared as a wide peak at 3000–3600 cm^−1^. The peak at 1605 cm^−1^ shows the presence of the carbonyl groups in the final composite structure, due to the presence of the CMC in the final composite.

To determine the formation and crystal structure of the materials, the XRD patterns of the CMC/PVA/SBA-15–NH_2_@AgNPs film, containing SBA-15–NH_2_ materials containing 3, 7, and 10 percent of Ag NPs are provided in Fig. 1b. All the XRD patterns show the wide characteristic peak of silica materials at around 2*θ* = 20°. This peak proves the presence of the SBA-15 materials in all three films. Due to the overlap of the characteristic peak of the CMC–PVA with the broad peak of the SBA-15, no new peak regarding CMC–PVA has appeared in this pattern. However, none of the present XRD patterns show the characteristics of the Ag NPs due to the low concentration of the NPs in the final composite. As an indication of the formation of the Ag NPs with the proposed method, the XRD pattern of the free Ag NPs, reduced using *Euphorbia* plant extract, is provided in Fig. S1,† showing the formation of the Ag NPs.

Thermogravimetric analysis (TGA) is conducted to study the thermal resistance of the composite. Fig. 1c, shows the TGA plots of the CMC/PVA/SBA-15–NH_2_@AgNPs film, containing SBA-15–NH_2_ materials containing 3, 7, and 10 percent of Ag NPs. This data shows good thermal stability up to nearly 300 °C for all three samples. The TGA plot shows a small weight loss in the first 100 °C, which is due to the loss of surface moisture and dissolved gasses in the film matrix. A sharp weight loss is accrued at 300–400 °C, regarding the loss of the organic content of the composite. The residues at 800 °C are the SBA-15 and the Ag NPs. This analysis shows that the increase in the Ag content of the composite resulted in a higher amount of inorganic residue, proving the formation of the composite as it was suggested. Also, low inorganic residue shows the low loading of the SBA-15–NH_2_@AgNPs into the film structure which is the reason for the absence of the characteristic peaks of Ag NPs in the XRD patterns.

**Fig. 2:**
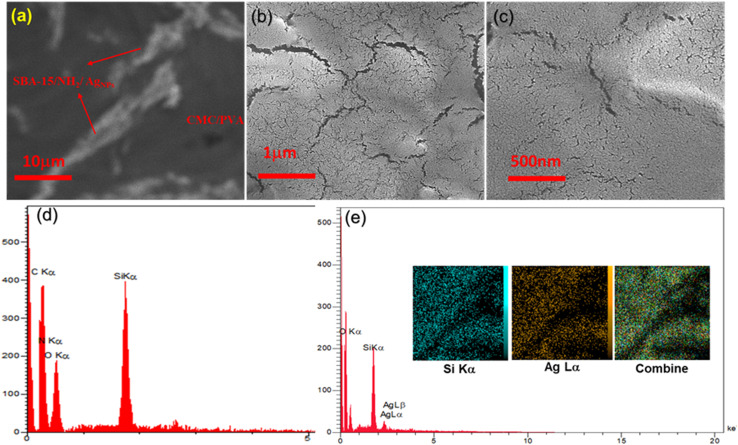
(a) The HADAF image of the CMC/PVA/SBA-15–NH_2_@AgNPs, (b and c) the SEM images of CMC/PVA/SBA-15–NH_2_ and CMC/PVA/SBA-15–NH_2_@AgNPs films, (d and e) EDX analysis of the CMC/PVA/SBA-15–NH_2_ and CMC/PVA/SBA-15–NH_2_@AgNPs films, respectively (the inset images are the elemental mapping images of the CMC/PVA/SBA-15–NH_2_@AgNPs film).

The HADAF images of the final composite is represented in Fig. 2a, which clearly shows the special dispersion of the SBA-15–NH_2_@AgNPs in the CMC/PVA matrix. The morphology and the surface structure of the composite are studied by the SEM technique. As shown in Fig. 2b, the worm-like roughness over the surface of the CMC–PVA film shows the formation of the SBA-15 materials in the CMC–PVA film matrix. The SEM image of CMC/PVA/SBA-15–NH_2_@AgNPs film also shows the presence of the SBA-15, but no indication of the presence of the Ag NPs is exhibited in this image, which proves the formation of the Ag NPs in the pores of the SBA-15 (Fig. 2c).

The composition and the elemental special distribution of the material is investigated by the EDX and SEM-mapping analysis (Fig. 2d and e). The results of this study indicate the presence of Si, C, O, N, and Ag in the structure of the final composite, proving the purity and the formation of the materials. This data proves the presence of Ag in the final composite.

### 3.1. Bacteria isolated from patients with SSIs

From 20 bacteria isolated from patients with surgical wound infection, 14 (70%) *Staphylococcus aureus*, 5 (25%) *E. coli*, and 1 (5%) *Pseudomonas aeruginosa* bacteria were isolated. In our study, 6 (42.85%) *Staphylococcus aureus* were resistant to methicillin, which results were somewhat similar to previous studies.^2,24^

### 3.2. Antibacterial efficacy and inhibition of biofilm formation

Results from comparing the antibacterial effects of nanofibers consisting of different contents of silver nanoparticles, along with ciprofloxacin antibiotics, are presented in Table 1. According to these results, nanofibers with various silver nanoparticle percents demonstrated antibacterial effects against *Staphylococcus aureus* and *E. coli* isolates. The sensitivity was dose-dependent. Notably, nanofibers with 7% and 10% AgNP concentrations exhibited higher antibacterial effects than the 3% concentration (*P* < 0.05). On the other hand, the difference between the 7% and 10% concentrations was insignificant (*P* > 0.05). Previous studies on CMC/PVA nanofibers with different nanoparticle contents support our results.^24–26^ Despite the sensitivity of *Staphylococcus aureus* isolates to the ciprofloxacin antibiotic, MRSA isolates were resistant to this antibiotic. In comparison to ciprofloxacin, CMC/PVA nanofibers containing silver nanoparticles showed good antibacterial effects against MRSA, with higher effects observed at 7% and 10% nanoparticle concentrations (*P* < 0.05). Also, among *E. coli* isolates, although 2 isolates were resistant to ciprofloxacin, they showed good sensitivity to nanofibers. Generally, it is suggested that CMC/PVA/AgNP nanofibers (3% and 7% AgNP) could be used as a potential antibiotic against *Staphylococcus aureus*, especially MRSA, which is a common cause of surgical wound infections.

**Table 1:** Average inhibition zone for all prepared samples compared with ciprofloxacin as a positive control^a^

Bacterial isolates	Inhibition zone (mm)			
	CMC/PVA/SBA-15–NH_2_@AgNPs			D
	A	B	C	
MSSA 1	10.3 ± 1.5	16 ± 1	17.6 ± 1.15	21 ± 0.47
MSSA 2	10 ± 1	16.6 ± 0.57	18.6 ± 2.5	21.3 ± 1.25
MSSA 3	10.6 ± 0.58	15.6 ± 1.53	17.6 ± 0.58	22.6 ± 1.15
MSSA 4	11.6 ± 1.5	15.3 ± 0.58	16.6 ± 0.58	21.6 ± 1.25
MSSA 5	11.6 ± 0.58	15.3 ± 1.15	17 ± 2	22.3 ± 1.25
MSSA 6	9.6 ± 1.15	15 ± 1	15.6 ± 1.53	21.3 ± 0.8
MSSA 7	9.6 ± 1.7	15.3 ± 0.58	17.6 ± 1.15	22.3 ± 0.8
MSSA 8	9.6 ± 1.73	15.6 ± 0.58	17 ± 1	10 ± 0.8^R^
MRSA 9	9.6 ± 0.58	16.3 ± 0.58	17.6 ± 1.53	9 ± 0.8^R^
MRSA 10	9.6 ± 0.58	16.3 ± 2.1	18.6 ± 0.58	8.6 ± 0.47^R^
MRSA 11	9.6 ± 1.15	16.6 ± 1.52	18.6 ± 1.53	8 ± 0.8^R^
MRSA 12	9.6 ± 0.58	15 ± 1	16.3 ± 1.53	1.6 ± 2.35^R^
MRSA 13	9.6 ± 1.7	16.3 ± 0.58	17.3 ± 2	0^R^
MRSA 14	9.6 ± 1.15	17.3 ± 0.58	19.6 ± 0.58	0^R^
*E. coli* 1	8.3 ± 0.58	13.6 ± 0.58	15.3 ± 1.15	27 ± 1
*E. coli* 2	8.6 ± 1.15	14 ± 1	15 ± 1	27.3 ± 0.58
*E. coli* 3	8.3 ± 1.15	14	16.6 ± 0.58	27.3 ± 1.15
*E. coli* 4	8	13.6 ± 0.58	16	0^R^
*E. coli* 5	7.6 ± 1.15	13.3 ± 1.5	15.6 ± 1.15	0^R^
*P. aeruginosa*	10 ± 0.58	16 ± 1	19 ± 1.5	26 ± 0.58

a (A) AgNPs 3%, (B) AgNPs 7%, (C) AgNPs 10%, (D) ciprofloxacin as a positive control, MSSA; methicillin-sensitive *Staphylococcus aureus*, MRSA; methicillin-resistant *Staphylococcus aureus*, R; resistant to ciprofloxacin.

The anti-biofilm effects of nanofibers on bacteria were assessed using the microtiter plate method. The results indicate that nanofibers with different silver nanoparticle concentrations effectively inhibited biofilm formation in both Gram-positive (*Staphylococcus aureus*; ATCC-43300) and Gram-negative (*Pseudomonas aeruginosa*; ATCC-27853) bacteria. The results suggest that these nanofibers could have medical applications (in the production of prosthetic coatings and medical equipment) and industrial applications (food packaging).

### 3.3. Toxicity of the CMC/PVA/SBA-15–NH_2_@AgNPs

**Fig. 3:**
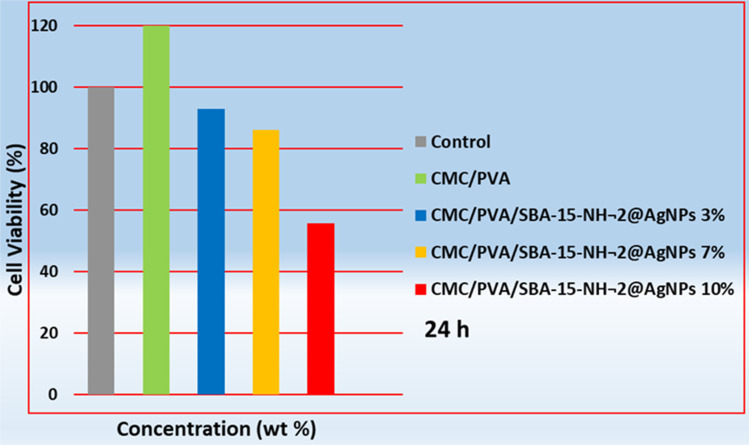
Cell viability of fibroblast cells (NHDF) exposed to PVA/CMC and CMC/PVA/SBA-15–NH_2_@AgNPs with different content of silver nanoparticles (3, 7, and 10 wt%). The viability of the control cells was set at 100%.

Using the MTT method, cytotoxicity testing was performed. Fig. 3 shows the survival of fibroblast cells exposed to nanofibers with different content of silver nanoparticles. The results displayed that CMC/PVA nanofibers while exhibiting no cytotoxicity, increased cell viability compared to the positive control group (*P* < 0.05). Consistent with previous studies, results indicated a transient decrease in cell viability with an increase in silver nanoparticle percentage (3% and 7%) within the nanofibers; however, this decrease was not statistically significant (*P* > 0.05). This study used a higher concentration of silver nanoparticles (3% and 7%) in the nanofibers compared to previous studies.^24,27^ Nevertheless, cell viability remained above 85%, while ultimately, cells exposed to nanofibers with 10% AgNP showed a significant decrease in viability compared to the two mentioned products (*P* < 0.05). The results suggest that CMC/PVA/AgNP nanofibers (3% and 7% AgNP) with high biocompatibility can be used as potential dressings for the treatment and prevention of surgical wound infections.

## 4. Conclusion

We created an antibacterial SBA-15–NH_2_ decorated biocompatible Ag NPs loaded CMC/PVA film that was tested against Gram-positive and Gram-negative bacteria strains in this work. To disclose the structure of the produced film, FT-IR, XRD, SEM, EDS, and TGA methods were used. The fashioned film is appropriate for a wide range of bio-related applications because of its flexible structure. The CMC/PVA/SBA-15–NH_2_@AgNPs film was used as a bactericidal agent against pathogens (especially MRSA) isolated from surgical site infections, showing promising results.

## Data availability

All data generated or analyzed during this study are included in this published article.

## Conflicts of interest

There are no conflicts to declare.

## Supplementary Material

RA-014-D4RA07129H-s001
