# Supplementary material for: A biocompatible cellulose gum based CMC/PVA/SBA-15 film as a colloidal antibacterial agent against MRSA
Source: RSC Adv. 2024 Nov 13;14(49):36246–52. doi: 10.1039/d4ra07129h (PMC11559378; doi:10.1039/d4ra07129h)
Supplement: RA-014-D4RA07129H-s001 [file RA-014-D4RA07129H-s001.pdf]

Supplementary Information (SI) for RSC Advances.
This journal is © The Royal Society of Chemistry 2024

Supporting information

Biocompatible cellulose gum-based CMC/PVA/SBA-15 film as a colloidal antibacterial agent against MRSA

Shiva Pakzad, Reza Taghavi, Amir Hasanzadeh, Sadegh Rostamnia

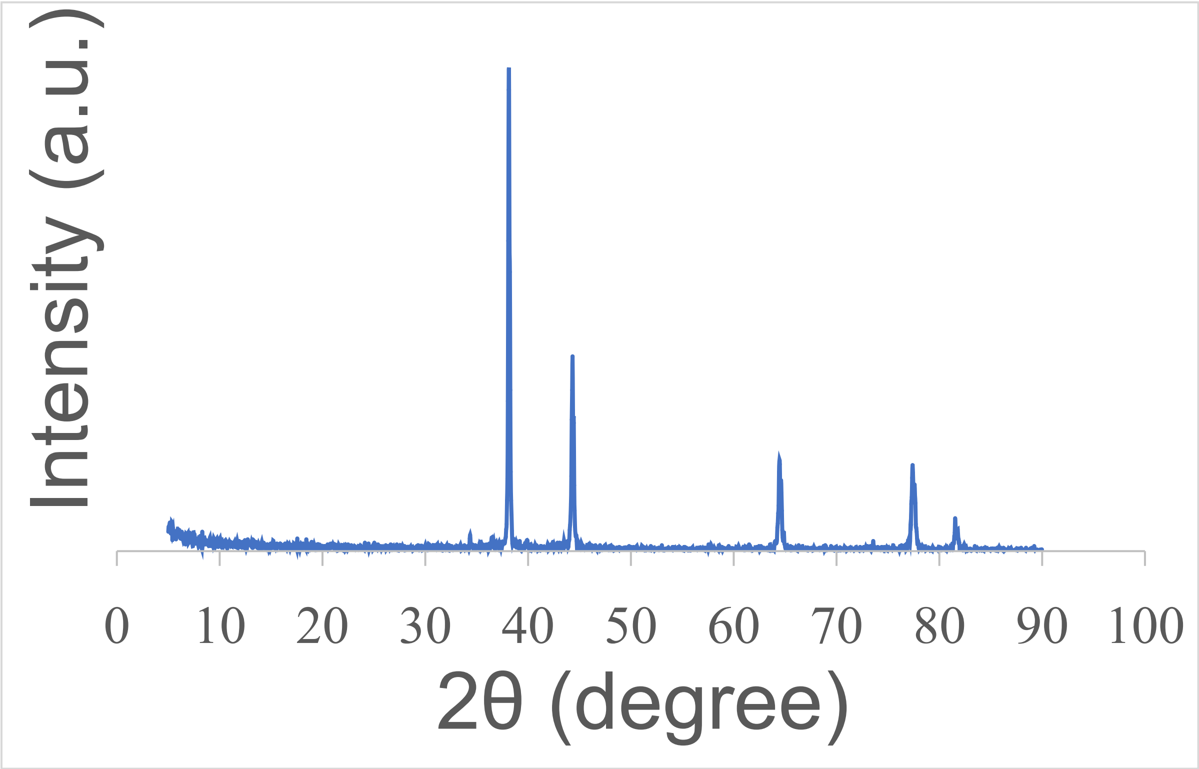

Fig. S1. The XRD pattern of the Free Ag NPs.
